# Comparative Analysis of the Long-Term Real-World Efficacy of Interleukin-17 Inhibitors in a Cohort of Patients with Moderate-to-Severe Psoriasis Treated in Poland

**DOI:** 10.3390/jcm14155421

**Published:** 2025-08-01

**Authors:** Wiktor Kruczek, Aleksandra Frątczak, Iga Litwińska-Inglot, Karina Polak, Zuzanna Pawlus, Paulina Rutecka, Beata Bergler-Czop, Bartosz Miziołek

**Affiliations:** 1 Students’ Scientific Association at the Department of Dermatology, School of Medicine in Katowice, Medical University of Silesia, 20/24 Francuska St., 40-067 Katowice, Poland; litwinska.iga@gmail.com (I.L.-I.);; 2 Department of Dermatology, School of Medicine in Katowice, Medical University of Silesia, 20/24 Francuska St., 40-067 Katowice, Polandbmiziolek@sum.edu.pl (B.M.)

**Keywords:** psoriasis, interleukin-17, bimekizumab, secukinumab, ixekizumab, Interleukin Inhibitors

## Abstract

**Background**: Bimekizumab, secukinumab, and ixekizumab are IL-17-targeting biologics approved for the treatment of moderate-to-severe plaque psoriasis. While secukinumab and ixekizumab selectively inhibit IL-17A, bimekizumab targets both IL-17A and IL-17F, potentially providing greater anti-inflammatory efficacy. This study aimed to compare the real-world effectiveness, safety, and tolerability of these agents in a Polish dermatology center between 2019 and 2024. **Methods**: We conducted a retrospective analysis of 98 patients meeting at least one of the following criteria: PASI ≥ 10, BSA ≥ 10, DLQI ≥ 10, or involvement of special areas with inadequate response or contraindications to ≥2 systemic therapies. Patients with prior exposure only to IL-17 inhibitors were excluded. PASI, BSA, and DLQI scores were recorded at baseline, week 4, and week 12. Due to differences in dosing schedules, outcomes were aligned using standardized timepoints and exponential modeling of continuous response trajectories. Mixed-effects ANOVA was used to assess the influence of baseline factors (age, BMI, PsA status) on treatment outcomes. Adverse events were documented at each monthly follow-up visit. **Results**: Bimekizumab showed the greatest effect size for PASI reduction (Hedges’ g = 3.662), followed by secukinumab (2.813) and ixekizumab (1.986). Exponential modeling revealed a steeper response trajectory with bimekizumab (intercept = 0.289), suggesting a more rapid PASI improvement. The efficacy of bimekizumab was particularly notable in patients who were previously treated with IL-23 inhibitors. All three agents demonstrated favorable safety profiles, with no serious adverse events or discontinuations. The most frequent adverse events were mild and included upper respiratory tract infections and oral candidiasis. **Conclusions**: This real-world analysis confirmed that IL-17 inhibitors effectively improved PASI, BSA, and DLQI scores in moderate-to-severe psoriasis. Bimekizumab demonstrated the most rapid early improvements and a higher modeled likelihood of complete clearance, without significant differences at week 12. All agents were well tolerated, underscoring the need for further individualized, large-scale studies.

## 1. Introduction

Plaque psoriasis, a chronic inflammatory skin disorder, significantly impacts the quality of life of affected individuals [1]. The condition is characterized by hyperproliferative keratinocytes, vascular alterations, and infiltration of immune cells, predominantly mediated by the dysregulated interplay of cytokines within the interleukin-23 and interleukin-17 axis [2]. Advances in biologic therapies targeting these pathways have transformed the therapeutic landscape for moderate-to-severe plaque psoriasis, offering unprecedented efficacy and safety profiles. Among the biologics approved for psoriasis treatment, bimekizumab, secukinumab, and ixekizumab have emerged as pivotal agents targeting IL-17 family cytokines [3]. Secukinumab and ixekizumab selectively inhibit IL-17A, a central cytokine in psoriatic inflammation [4]. Bimekizumab, however, represents a novel therapeutic approach by dual inhibition of IL-17A and IL-17F, which are both implicated in the pathophysiology of psoriasis [5]. The concurrent targeting of IL-17A and IL-17F by bimekizumab has been hypothesized to confer superior efficacy due to its broader suppression of the inflammatory cascade. Numerous investigations into psoriasis have consistently demonstrated a higher prevalence of comorbid conditions compared to the general population, including lipid metabolism disorders, obesity, arterial hypertension, type 2 diabetes, and hepatic dysfunction [6]. Particularly concerning is the increased risk of cardiovascular events, which contribute significantly to the reduced life expectancy observed in psoriasis patients—estimated to be approximately five years shorter than in unaffected individuals [7]. Myocardial infarction and thromboembolic events are identified as leading causes of mortality within this population, highlighting the urgent need for effective disease management [8]. These findings underscore the critical importance of timely and effective psoriasis treatment to mitigate systemic inflammation and its downstream complications [9]. Rapid disease control not only improves skin symptoms but may also reduce the burden of comorbidities, including cardiovascular risks, thereby enhancing overall patient health and longevity [10]. This study aims to provide a comparative analysis of the clinical efficacy, safety, and tolerability profiles of bimekizumab, secukinumab, and ixekizumab in the treatment of moderate-to-severe plaque psoriasis in the Polish population. Given the growing role of IL-17 inhibitors in dermatologic therapy and the expanding range of available agents, a better understanding of their comparative effectiveness and tolerability in routine clinical settings is essential. The present study aims to explore and compare the long-term efficacy, safety, and quality-of-life outcomes associated with secukinumab, ixekizumab, and bimekizumab in a real-world cohort of patients with moderate-to-severe plaque psoriasis treated within a national therapeutic program. In addition to reporting comparative clinical outcomes, this study applies an exploratory modeling approach using exponential functions to characterize continuous PASI, BSA, and DLQI trajectories over time. This analytic framework is intended to support a more nuanced understanding of treatment dynamics and may offer practical insights into individualized therapeutic monitoring.

## 2. Materials and Methods

A total of 98 patients (67 males, 68.4%) were included (mean age: 45.9 ± 13.26 years; BMI: 29.8 ± 5.21 kg/m^2^; disease duration: 20.5 ± 11.4 years), with 25.5% presenting concomitant psoriatic arthritis (Table 1). Patients in the long-term study were treated with IL-17 inhibitors as part of the B-47 national drug program at the Dermatology Clinic in Katowice between January 2019 and December 2024.

A retrospective analysis of the applied treatment focused on the parameters PASI, BSA, and DLQI in patients who underwent therapy with bimekizumab, secukinumab, and ixekizumab. PASI combines the extent of skin involvement and the severity of erythema, infiltration, and scaling across four body regions, yielding a score from 0 to 72. BSA was estimated as the percentage of total body surface affected by psoriatic lesions, using standard dermatological estimation methods (e.g., the palm rule). DLQI is a validated 10-item questionnaire assessing the impact of skin disease on the patient’s quality of life, with scores ranging from 0 (no impairment) to 30 (maximum impairment). All assessments were performed by experienced dermatologists at scheduled follow-up visits in line with the national therapeutic protocol. Patients were consecutively enrolled based on eligibility for IL-17 inhibitor therapy under the B-47 national therapeutic program. All individuals who initiated treatment between January 2019 and December 2024 at the Dermatology Clinic in Katowice and met the inclusion criteria were retrospectively included in the analysis. The severity of psoriasis meets the following indices: PASI ≥ 10, BSA ≥ 10, and DLQI ≥ 10. Thresholds were not required to be met simultaneously. In addition, the inclusion criteria involved the following special areas: the scalp, face, genital area, hands, feet, nails, and a lack of response to or contraindications for at least two different conventional systemic therapies. Patients were excluded from the study if they had previously received interleukin-17 inhibitors as part of their therapy. Additionally, women who were pregnant or breastfeeding were not eligible for participation. Individuals with hypersensitivity to the active ingredient or any excipients of the drug were disqualified. The presence of active or latent infections, as well as active malignancies or a history of cancer treatment completed within the last five years, constituted further exclusion criteria. Finally, patients diagnosed with pancytopenia or aplastic anemia were also deemed ineligible for the study. Only patients with complete clinical records at the predefined assessment timepoints (weeks 0, 4, and 12) were included in the final analysis. No data imputation was performed. Clinical assessments of treatment efficacy and adverse events were conducted during monthly follow-up visits throughout the entire course of therapy, with each patient participating in the study for a minimum duration of 12 weeks.

Patients were administered bimekizumab in accordance with the prescribing information, which specifies a dosage of 320 mg delivered as two subcutaneous injections of 160 mg each. The treatment regimen includes doses at weeks 0, 4, 8, 12, and 16, followed by maintenance injections every 8 weeks thereafter. The recommended dosing regimen for ixekizumab begins with a loading dose of 160 mg administered as two subcutaneous injections of 80 mg each at week 0. This is followed by a maintenance phase with a dose of 80 mg (one injection) administered at weeks 2, 4, 6, 8, 10, and 12. Subsequently, a maintenance dose of 80 mg is administered every 4 weeks. The dosing regimen for secukinumab consists of 300 mg administered via subcutaneous injection. During the initial phase, the medication is given at weeks 0, 1, 2, 3, and 4. This is followed by a maintenance phase with monthly doses. Each 300 mg dose is delivered as two subcutaneous injections of 150 mg each. Due to differences in the dosing regimens of IL-17 inhibitors, analyses of therapeutic efficacy parameters were conducted continuously and at common dosing points for all three drugs, namely: day 1 of therapy, week 4 (corresponding to the administration of the second dose of bimekizumab, the third dose of ixekizumab and the fifth dose of secukinumab) and week 12 of therapy. To evaluate whether baseline demographic and clinical differences influenced treatment outcomes, we conducted additional statistical analyses, including mixed-effects ANOVA models and corrected correlation tests. These analyses explored the impact of age, sex, BMI, and psoriatic arthritis (PsA) status on changes in PASI, BSA, and DLQI across treatment groups. Safety and tolerability were evaluated during the follow-up of the study. Adverse events experienced by the patients were reported. The collected data were subjected to statistical analysis. No data imputation was performed. The analyses were performed using the Python programming language (version 3.11.8) along with the following libraries: Pandas (2.2.1) for data processing, Matplotlib (3.8.3) and Seaborn (0.13.2) for data visualization, and Pingouin (0.5.4) for statistical computations. A *p*-value of <0.05 was considered statistically significant.

## 3. Results

In total, there were 98 patients divided into study groups based on applied treatment: 48 patients with bimekizumab, 20 patients with ixekizumab, and 30 patients with secukinumab.

**Figure 1:**
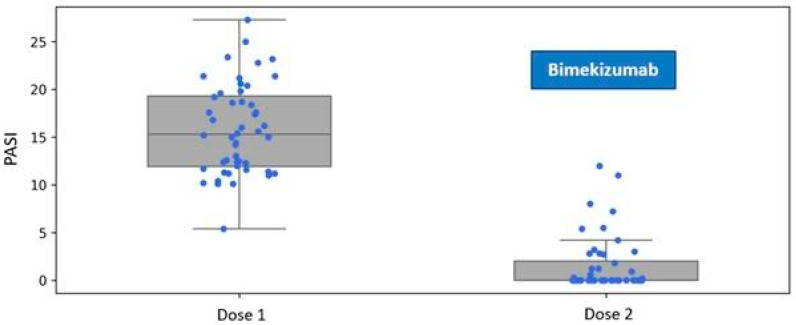
Reduction in the Psoriasis Area and Severity Index (PASI) following a single dose of bimekizumab, assessed at the one-month follow-up visit during biological therapy.

Patients assigned to the Bimekizumab treatment group exhibited a mean disease severity score of PASI 15.84 (SD 4.72) at baseline. One month after the administration of the first dose, during the follow-up visit for the second dose, the mean severity of psoriatic lesions decreased to 1.54 (SD 2.89) (Figure 1).

**Figure 2:**
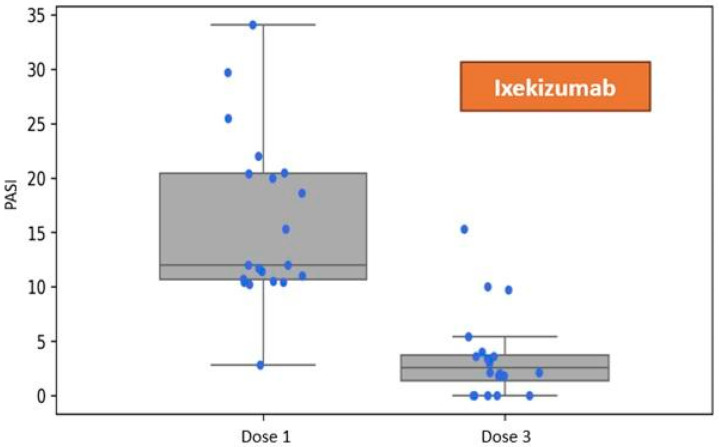
Reduction in the Psoriasis Area and Severity Index (PASI) following three doses of ixekizumab, assessed at the one-month follow-up visit during biological therapy.

Patients assigned to the group receiving Ixekizumab exhibited a mean disease severity of PASI 15.96 (SD 7.71) at baseline. One month following the administration of the first dose, during the visit corresponding to the administration of the third dose per the dosing schedule, the mean severity of skin lesions was recorded at 3.54 (SD 3.96). Refer to Figure 2.

**Figure 3:**
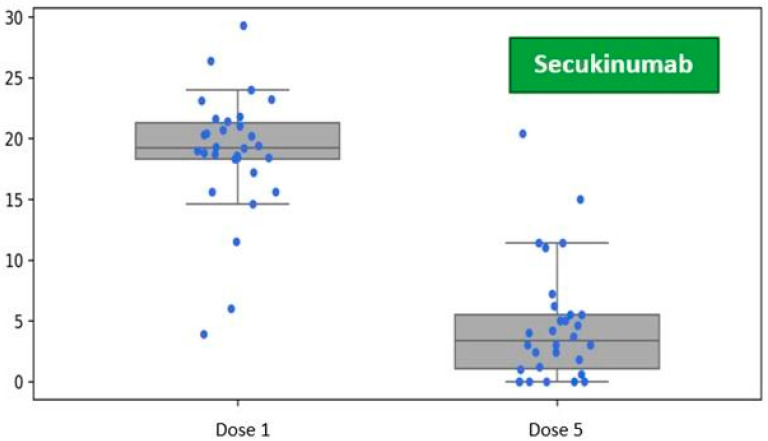
Reduction in the Psoriasis Area and Severity Index (PASI) following five doses of secukinumab, assessed at the one-month follow-up visit during biological therapy.

Patients in the secukinumab treatment group demonstrated an average baseline disease severity of PASI 18.86 (SD 5.10). One month after receiving the initial dose, during the visit for the fifth dose administration as per the prescribed schedule, the average severity of skin lesions decreased to PASI 4.62 (SD 4.90) (Figure 3).

A comparative analysis of the efficacy of IL-17 inhibitors in reducing the PASI was conducted using the paired-sample *t*-test, demonstrating that the changes observed after one month of treatment with bimekizumab, ixekizumab, and secukinumab were statistically significant. The effect size, expressed as Hedges’ g, quantifies the reduction in PASI values in terms of standard deviation units. Bimekizumab achieved the Hedges’ g value of 3.662, indicating clinical efficacy in PASI reduction among the evaluated IL-17 inhibitors, corresponding to a mean decrease of 3.662 standard deviations. Secukinumab followed with a Hedges’ g value of 2.813, and ixekizumab with a value of 1.986. Refer to Table 2 for details.

**Figure 4:**
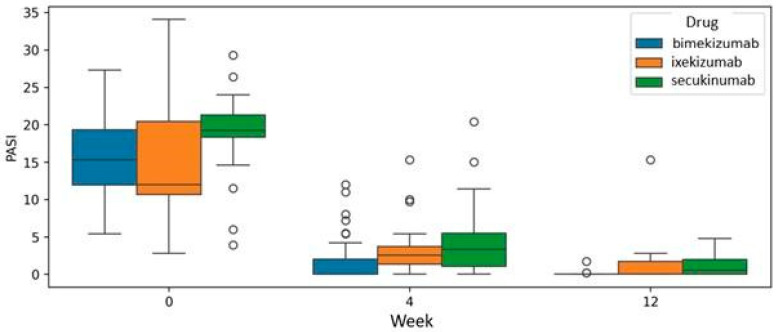
Efficacy comparison of bimekizumab, ixekizumab, and secukinumab in reducing Psoriasis Area and Severity Index (PASI) scores at the common treatment endpoint of week 12. Analysis was performed using a mixed model analysis of variance (mixed ANOVA).

The analysis at the common endpoint of treatment with all three drugs, specifically at week 12, was conducted using a mixed model analysis of variance (mixed ANOVA). Bimekizumab, ixekizumab, and secukinumab demonstrated efficacy in reducing PASI scores. However, the mixed ANOVA did not reveal a statistically significant difference (*p* = 0.359), indicating a superior drug at week 12 of therapy (Figure 4).

To visualize trends beyond fixed assessment points, we constructed a scatter plot of mean PASI values over time and applied an exponential interpolation function to model the continuous therapeutic trajectory. In this exploratory analysis, the intercept of the fitted exponential curve was used as a comparative indicator of proximity to PASI100, with lower values suggesting a steeper decline in disease severity. Bimekizumab exhibited the lowest intercept (0.289), followed by ixekizumab (1.45) and secukinumab (1.55), indicating a numerically greater tendency toward complete clearance in the bimekizumab group. These findings, however, should be interpreted with caution, as the intercept is not a validated clinical endpoint and does not account for individual variability or potential confounders.

**Figure 5:**
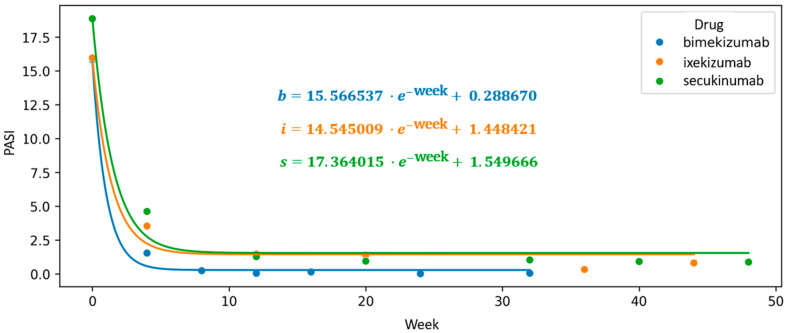
Trajectory of long-term biologic treatment visualized through a scatter plot of mean Psoriasis Area and Severity Index (PASI) values, with an interpolated exponential function modeling the therapeutic response over time. The intercept of the fitted curve indicates its deviation from the *Y*-axis and reflects the theoretical PASI100 achievement threshold, providing insight into the expected timing and extent of maximal clinical response.

The first coefficient of the exponential function, associated with Euler’s number, reflects the rate of decline in mean PASI scores. While informative, its interpretation is limited by sensitivity to initial disease severity. This modeling approach is, therefore, intended to complement conventional statistical comparisons and to offer an exploratory perspective on treatment dynamics over time (Figure 5).

An exploratory analysis using exponential function interpolation was also applied to evaluate changes in the BSA parameter throughout the course of continuous biologic therapy. Among the agents studied, bimekizumab displayed the steepest initial slope of the function (a = 27.714), indicating a more rapid early reduction in body surface area affected by psoriasis. Additionally, it showed the lowest intercept value (c = 0.287), which, within the framework of this model, may reflect a more favorable trajectory toward complete skin clearance (BSA = 0).

**Figure 6:**
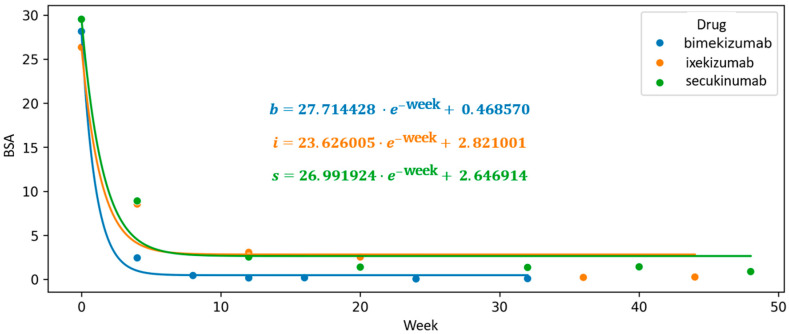
Longitudinal course of biological therapy visualized by a scatter plot of mean Body Surface Area (BSA) values, with an interpolated exponential function modeling the treatment trajectory. The intercept of the curve reflects its deviation from the *Y*-axis and corresponds to the theoretical achievement of complete skin clearance (BSA = 0). The exponential interpolation highlights the dynamic reduction in BSA over time during continuous biologic treatment.

In comparison, higher intercept values were observed for secukinumab (c = 2.647) and ixekizumab (c = 2.821), corresponding to a relatively less pronounced modeled trajectory. These results, while indicative of differing response dynamics between agents, should be interpreted with caution, as the model parameters are not validated predictors and serve primarily to support comparative visualization of treatment trends (Figure 6).

**Figure 7:**
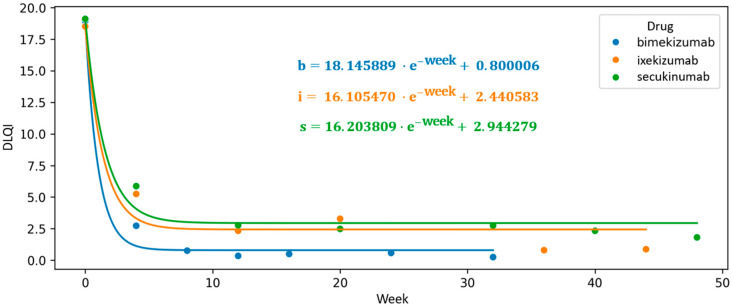
Long-term assessment of quality of life in patients receiving IL-17 inhibitor therapy, illustrated by a scatter plot of mean Dermatology Life Quality Index (DLQI) scores and an interpolated exponential function modeling the full treatment trajectory. The function’s intercept indicates the curve’s deviation from the *Y*-axis, corresponding to the ideal outcome of DLQI = 0.

Continuous monitoring of DLQI throughout the treatment course allowed for a comprehensive visualization of sustained improvements in patient-reported outcomes during biological therapy. Based on the collected data, we applied exponential function interpolation to illustrate the continuity of changes observed in patients undergoing treatment with IL-17 inhibitors. The intercept value of the function for patients undergoing bimekizumab therapy is the lowest, at 0.8, indicating the highest likelihood of achieving a Dermatology Life Quality Index (DLQI) score of 0. This score signifies a complete absence of any impact of a dermatological condition on the patient’s quality of life, reflecting no interference with physical, emotional, or social functioning. Furthermore, bimekizumab therapy was associated with the most rapid initial decline in DLQI with a function coefficient of a = 18.146. In contrast, ixekizumab and secukinumab demonstrated statistically lower capacities for complete DLQI reduction in the studied patient population with intercept values of c = 2.441 and c = 2.944, respectively (Figure 7).

Additional analyses to assess the potential influence of baseline demographic and clinical variables on treatment outcomes across all therapeutic groups showed a significant inverse correlation observed between age and BSA improvement between doses 2 and 3 in the bimekizumab group (r = −0.47, *p* = 0.001; *p*-corrected = 0.002). Additionally, subjective DLQI improvement over time was significantly influenced by sex in the ixekizumab group (interaction *p* = 0.022), and by PsA status in the secukinumab group (interaction *p* = 0.028). However, no consistent confounding effects of baseline demographics were found across biologics. Treatment-related improvement remained the strongest effect in all models (η^2^ > 0.72). Full results are presented in Supplementary Table S1.

**Figure 8:**
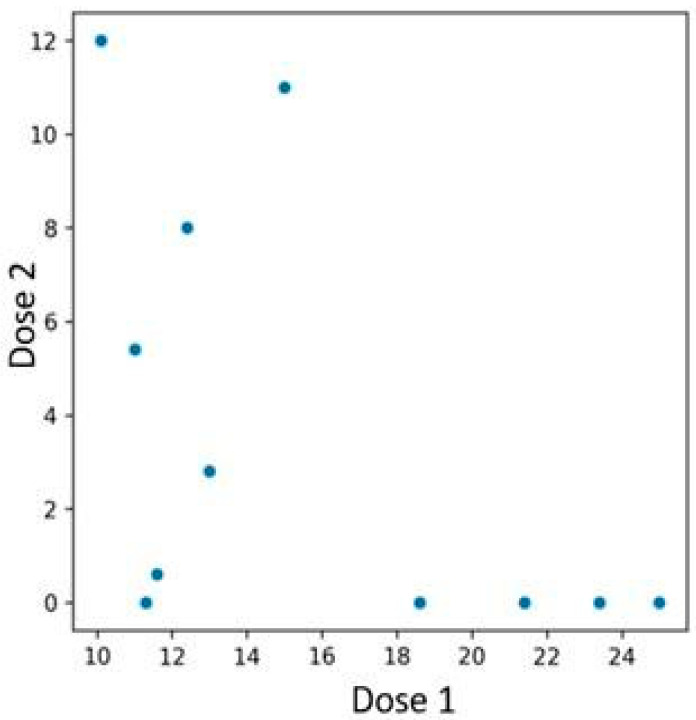
Relationship between PASI scores at the time of first and second bimekizumab administration, with subgroup analysis based on prior IL-23 inhibitor exposure. Patients with higher baseline PASI values demonstrated a more rapid response to bimekizumab, whereas those with lower initial scores showed a more gradual decline.

Among the 48 patients treated with bimekizumab, 12 individuals who had previously been treated with an interleukin-23 inhibitor were identified. Patients previously treated with an IL-23 inhibitor experienced a more rapid PASI reduction if their baseline PASI score before initiating bimekizumab therapy was higher (Figure 8). In contrast, those with lower initial PASI values exhibited a more gradual reduction. The performed Spearman correlation test confirmed the presence of the described association, yielding a *p*-value of 0.019.

**Figure 9:**
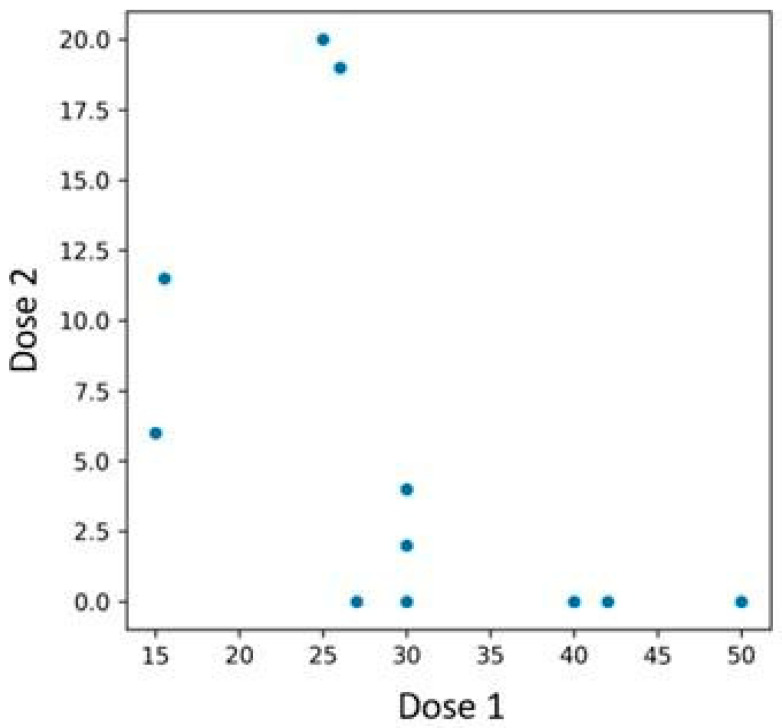
Relationship between BSA scores at the time of first and second bimekizumab administration, with subgroup analysis based on prior IL-23 inhibitor exposure.

A similar pattern was observed in BSA reduction—patients with lower baseline BSA experienced a less pronounced decrease compared to those with higher initial values (Figure 9). The Spearman correlation test was conducted, yielding a *p*-value of 0.003.

In this original study, long-term patient follow-up revealed only a limited number of adverse effects associated with IL-17 inhibitors. Among patients treated with bimekizumab, upper respiratory tract infections were observed in two cases, and a single case of oral candidiasis was reported. In the ixekizumab group, three patients experienced urticaria and injection site reactions, while one patient developed oral candidiasis. In the secukinumab-treated cohort, upper respiratory tract infections were documented in three individuals. All reported adverse events were classified as mild and resolved without complications following local symptomatic treatment. Detailed information on adverse events associated with IL-17 inhibitors observed in the study cohort is presented in Table 3.

## 4. Discussion

Psoriasis is a multifactorial, immune-mediated disease. While its complex pathogenesis involving IL-23/IL-17 cytokine axes is well described, this study aimed to examine not mechanisms but real-world treatment dynamics in a Polish cohort receiving IL-17-targeted biologics.

The primary objective of psoriasis treatment is to achieve and sustain comprehensive lesion clearance over the long term while concurrently managing chronic systemic inflammation and preventing the onset and progression of associated systemic comorbidities. The choice of treatment is dependent upon the disease’s severity, which is influenced by factors such as the extent of the lesions, their anatomical location, the level of inflammation, and the resultant impact on the patient’s quality of life.

The advent of biologic therapies targeting pro-inflammatory cytokines has transformed the treatment paradigm for moderate-to-severe plaque psoriasis [11]. In particular, IL-17 inhibitors have emerged as one of the most potent therapeutic options currently available [12]. The three main IL-17 inhibitors used in clinical practice—secukinumab, ixekizumab, and bimekizumab—offer robust efficacy and generally favorable safety profiles, yet they differ in structure, mechanism, pharmacodynamics, and clinical outcomes [13].

### 4.1. Therapeutic Goal

The Psoriasis Area and Severity Index (PASI) remains a pivotal tool in the evaluation of disease severity and treatment response in patients with psoriasis. Historically, achieving a PASI 75 response—indicating a 75% reduction from baseline PASI scores—represented a significant therapeutic milestone [14]. However, with recent advancements in biologic therapies and targeted immunomodulators, clinical expectations have shifted toward more ambitious endpoints, such as PASI 90 and even PASI 100 responses, the latter indicating complete skin clearance [15].

These higher response thresholds are increasingly recognized as optimal therapeutic outcomes in both clinical trials and real-world settings. Importantly, such outcomes align not only with improved clinical status but also with enhanced patient-reported outcomes and quality of life. Given the heterogeneous nature of psoriasis—with interindividual variability in lesion morphology, anatomical distribution, disease chronicity, and severity—therapeutic strategies must be tailored accordingly. Moreover, psoriasis exerts a profound impact beyond the physical domain, affecting psychological well-being, social integration, occupational performance, and intimate relationships [16].

Hence, management should be patient-centric, integrating shared decision-making to establish individualized, realistic treatment goals. Evidence supporting this approach is further reinforced by economic analyses. In a study by Maravilla-Herrera et al., the estimated annual cost per patient achieving PASI 75 was EUR 6139. In contrast, patients reaching PASI 90 and PASI 100 demonstrated significantly lower economic burdens, estimated at EUR 3956 and EUR 1353, respectively [17].

These findings highlight that greater therapeutic efficacy translates into lower societal costs—through reduced healthcare utilization, improved productivity, and diminished out-of-pocket expenditures. Thus, striving for complete skin clearance may confer not only superior patient outcomes but also broader economic and societal benefits.

There is a recognized need for the implementation of real-world studies.

The interpretability of clinical trial outcomes in psoriasis is limited by stringent inclusion and exclusion criteria, often leading to homogenous study populations that poorly represent the heterogeneous nature of real-world patients. This selection bias restricts the external validity of trial findings, particularly for individuals with comorbidities, prior biologic exposure, or complex treatment histories. Real-world studies (RWS) address this limitation by evaluating therapeutic outcomes in more diverse and clinically relevant populations [18]. By including older adults, polypharmacy patients, and those with multimorbidity, RWS offers pragmatic insights into treatment effectiveness, safety, and tolerability that complement trial data. However, their retrospective design can reduce methodological rigor and reproducibility. Thus, RWS should not substitute for randomized trials but rather augment them. An integrated approach combining data from both settings enhances clinical relevance, facilitating more nuanced and patient-centered decision-making in routine dermatological care [19].

### 4.2. Ixekizumab and Secukinumab

Secukinumab and ixekizumab are both biologic agents that target IL-17A. Head-to-head trials have shown that ixekizumab and secukinumab provide greater clinical efficacy in treating psoriasis compared to established therapies like ustekinumab (an anti-IL-12/23 antibody) and etanercept (a TNF receptor inhibitor) [20]. They differ in their antibody subclasses—secukinumab is an IgG1 monoclonal antibody, while ixekizumab belongs to the IgG4 subclass [21]. IgG4 is thought to be less immunogenic than IgG1 molecules, because it does not activate the classic complement system [22]. Moreover, pharmacokinetic differences are clinically relevant. Secukinumab reaches steady-state concentrations later (after 20 weeks), which may influence peak clinical efficacy [23,24]. In contrast, ixekizumab reaches peak levels faster, offering more rapid symptom relief—a key factor for patients with severe or psychologically burdensome disease [25].

In our exploratory modeling of long-term treatment response, ixekizumab and secukinumab showed closely comparable efficacy trajectories. Based on PASI score reduction, ixekizumab exhibited a slightly steeper modeled decline, which may suggest a higher likelihood of approaching PASI100. However, when examining predicted BSA trajectories, secukinumab showed a marginal advantage in reaching complete skin clearance (BSA = 0). The analysis of DLQI (Dermatology Life Quality Index) reduction indicated a superiority of ixekizumab over secukinumab. This finding is likely associated with differences in the dosing regimens of the two agents. Specifically, during the induction phase, secukinumab is administered via subcutaneous injection on a weekly basis, whereas ixekizumab is administered biweekly. A key concern in chronic immunomodulatory treatment is the emergence of anti-drug antibodies (ADAs), which may neutralize drug activity or accelerate clearance, potentially leading to secondary loss of response [26]. Importantly, while ixekizumab may lead to the development of anti-drug antibodies up to 1.7–2.4%, these rarely impact clinical efficacy. Conversely, secukinumab exhibits minimal immunogenicity (0–0.41% ADA incidence), possibly due to its fully human antibody structure [11]. Ongoing pharmacovigilance efforts and long-term patient registries are critical for thoroughly evaluating the durability of therapeutic efficacy and assessing the potential risk of treatment attenuation due to the development of anti-drug antibodies.

### 4.3. Bimekizumab

Bimekizumab, a monoclonal IgG1 antibody, represents a novel therapeutic approach by simultaneously neutralizing both IL-17A and IL-17F. This dual specificity is achieved through its antigen-binding sites, which are identical on each arm and capable of optimally binding either cytokine, thereby preventing pro-inflammatory signaling regardless of their relative concentrations [27]. The lack of statistical significance in the mixed ANOVA model conducted at week 12 did not demonstrate superiority of bimekizumab over secukinumab or ixekizumab at this shared endpoint. However, it is critical to contextualize this finding within the broader analytical framework and temporal structure of the study. The mixed ANOVA at week 12, while methodologically appropriate for cross-sectional comparison, captures only a single timepoint and does not reflect the trajectory or dynamics of therapeutic response, which is particularly relevant in real-world psoriasis treatment. To address this, our study complemented classical hypothesis testing with exponential modeling of PASI, BSA, and DLQI trajectories, enabling longitudinal estimation of response kinetics. These models demonstrated that bimekizumab had the lowest intercepts across all outcomes—indicating a higher probability of achieving complete disease clearance (PASI100, BSA = 0, DLQI = 0). Moreover, its early effect size (Hedges’ g = 3.662) far exceeded those of ixekizumab (1.986) and secukinumab (2.813), suggesting a more rapid and consistent early response, corroborated by significant within-group PASI improvements from baseline to week 4 (*p* < 0.001). Therefore, while the week 12 comparison did not reach between-group significance, this isolated timepoint does not negate the overall superiority trend observed in longitudinal analyses. These findings are consistent with clinical trial data, indicating superior PASI rates for bimekizumab over secukinumab at both 16 and 48 weeks [28]. While secukinumab and ixekizumab both showed substantial reductions in disease severity, a study conducted by Kokolakis G et al. revealed a greater likelihood of complete skin clearance (PASI100) and normalization of quality of life (DLQI = 0) with bimekizumab, underscoring the clinical benefit of dual IL-17A/F inhibition in achieving long-term remission [29].

### 4.4. Comparative Perspective Across European Real-World Cohorts

The originality of our study lies in its exclusive focus on a Polish cohort of patients treated within a national therapeutic program, providing valuable real-world insights into the effectiveness and safety of IL-17 inhibitors in a Central European population. To place our findings into a broader context, it is important to compare them with data from other European registries, particularly those capturing long-term outcomes in similar clinical settings.

In this regard, a large real-world analysis from the Czech BIOREP registry [30] offers a meaningful comparison. The study included 949 patients treated with brodalumab, ixekizumab, or secukinumab, of whom 275 received ixekizumab and 490 received secukinumab. Unlike our study, the Czech cohort comprised both biologic-naïve and previously treated patients, reflecting a more heterogeneous clinical population. Over a 24-month period, ixekizumab demonstrated superior long-term efficacy compared to secukinumab, with PASI90 and PASI100 response rates of 74.4% and 46.7%, respectively, versus 65.0% and 41.6% for secukinumab. Furthermore, discontinuation due to loss of efficacy was more frequent with secukinumab (22.0%) than with ixekizumab (13.1%), despite similar baseline PASI and DLQI scores across treatment groups.

By contrast, our analysis, which excluded patients previously treated with IL-17 inhibitors, showed a greater early effect size (Hedges’ g) for secukinumab (2.813) compared to ixekizumab (1.986) after one month of therapy. This may reflect the pharmacokinetic advantage of secukinumab in biologic-naïve patients with higher baseline PASI scores. However, long-term projections based on exponential modeling of PASI trajectories in our cohort suggested a slightly higher likelihood of achieving PASI100 with ixekizumab (intercept = 1.45) versus secukinumab (intercept = 1.55). Additionally, improvements in Dermatology Life Quality Index (DLQI) scores were more favorable with ixekizumab, consistent with findings from the Czech registry.

These inter-cohort differences may be attributed to varying inclusion criteria (e.g., prior biologic exposure), treatment algorithms, population-level comorbidities (e.g., obesity, psoriatic arthritis), or healthcare system structures. Notably, in the Czech study, obesity and the number of previous biologic treatments were negative predictors of drug survival, whereas concomitant psoriatic arthritis was associated with better treatment persistence.

Other European populations, particularly the Swiss cohort analyzed by Lam et al. [31] in a recent single-center study, compared secukinumab and ixekizumab over a 10-year period in routine clinical practice.

The Swiss cohort included a smaller sample (*n* = 81); furthermore, the prevalence of psoriatic arthritis was considerably higher in the Swiss population (59.3%) compared to our cohort (25.5%), which may have influenced baseline disease severity, drug survival, and treatment selection.

From an outcome’s perspective, both studies observed higher initial and sustained PASI responses for ixekizumab over secukinumab; however, in our study, the efficacy of ixekizumab remained lower than that of bimekizumab, which demonstrated the most rapid and pronounced PASI, BSA, and DLQI reductions. In the Swiss analysis, ixekizumab showed numerically better PASI75 and PASI90 response rates at weeks 52 and 104 compared to secukinumab (PASI75 at week 52: 74.6% vs. 55.4%), but the difference was not statistically significant. Interestingly, in our cohort, both agents reached similar endpoints at week 12, as shown by a mixed ANOVA.

The Swiss study reported longer drug retention for ixekizumab versus secukinumab, with factors such as nail psoriasis and inverse psoriasis associated with longer persistence, whereas female sex and multiple prior therapies increased discontinuation risk. In contrast, our population showed uniformly high tolerability and low discontinuation rates for all IL-17 inhibitors, with adverse events being mild and self-limiting.

### 4.5. Previous Interleukin-23 Inhibitor Inefficacy

The switch to bimekizumab was due to treatment inefficacy rather than adverse effects. Consequently, the transition was made without a washout period, in accordance with the recommendations outlined in the review [32]. In patients with a history of IL-23 inhibitor treatment, initiation of bimekizumab therapy led to a more pronounced and accelerated reduction in PASI scores, particularly when baseline PASI levels were elevated. Conversely, individuals presenting with lower initial PASI scores demonstrated a more gradual therapeutic response. This phenomenon may reflect an alternative immunological activation cascade predominating in individuals exhibiting inadequate response to IL-23 blockade, where T-cell stimulation is primarily mediated by activated neutrophils rather than conventional antigen-presenting mechanisms. A comparable trend was evident with BSA metrics, where patients with higher initial BSA involvement showed a more substantial decline post-treatment compared to those with lower baseline values. Notably, the presence of neutrophil-driven IL-17 synthesis pathways, which are not effectively inhibited by IL-23-targeted agents, could contribute to the observed therapeutic discrepancies. Bimekizumab’s dual inhibition of IL-17A and IL-17F potentially disrupts these alternative inflammatory loops more efficiently, thereby explaining its superiority in such clinical contexts [33]. Further mechanistic studies involving tissue cytokine profiling and neutrophilic infiltration analysis are warranted to validate this hypothesis. From a translational perspective, stratifying patients based on prior biologic exposure and baseline inflammatory metrics may enable personalized therapeutic approaches. This would be especially pertinent for individuals with partial or suboptimal responses to IL-23 blockade, for whom bimekizumab may offer a more targeted and efficacious intervention [34].

### 4.6. Safety and Tolerability

Interleukin-17 inhibitors demonstrate good tolerance in patients and show positive safety results in both clinical trials and real-world patient data. The most frequent adverse effects reported by patients include upper respiratory tract infections. Common adverse events include headaches, sore throat, Candida infections, allergies, joint pain, high blood pressure, diarrhea, itching, and cough [35,36,37].

Serious adverse events occur rarely in treated patients and at low rates, which supports the extended safety of this therapeutic class. A study conducted by Berman J et al. shows a high safety profile for secukinumab and ixekizumab, as highlighted by the low rate of drug discontinuation due to adverse events [38].

The proportion of patients who developed oral candidiasis IL-17 therapy in our cohort may be partially explained by the relatively small sample size and the high prevalence of metabolic comorbidities, such as diabetes and obesity, which are recognized risk factors for candidiasis. It is important to note, however, that while oral candidiasis was observed, the causative link to psoriasis treatment cannot be definitively established, aligning with previous reports that some adverse events during long-term IL-17 inhibitor therapy may not be directly attributable to the drug itself. Moreover, the incidence of candidiasis decreased over time, the adverse event was generally mild and manageable, and no serious adverse events required treatment discontinuation. These findings further support the favorable long-term safety and tolerability profile in real-world clinical practice. The patients’ ability to continue therapy without treatment-limiting adverse effects, reflected by drug survival rates and patient-reported outcomes regarding treatment satisfaction.

### 4.7. Limitations

Although group sizes were unequal and baseline variability existed, additional analyses demonstrated that patient characteristics such as age, PsA coexistence, or sex did not systematically influence treatment response across biologics. Observed interactions were mostly related to DLQI, a subjective, patient-reported outcome inherently influenced by individual perception. Notably, objective measures like PASI and BSA remained largely unaffected by baseline differences. The only exception was a transient association between age and early BSA improvement in the bimekizumab group. These findings suggest that the differences in treatment outcomes observed in our cohort likely reflect true pharmacodynamic effects rather than bias from population heterogeneity.

While our sample size limits broad generalizability, this study presents an important step in integrating continuous-time modeling with real-world psoriasis management in a Central European population. The insights gained from the Polish cohort highlight both the strengths of IL-17 inhibition and the importance of regional treatment context. These findings underscore the need for larger multinational studies that incorporate dynamic modeling techniques to refine personalized treatment strategies globally.

The limitation of this study lies in the variation in dosing regimens between the IL-17 inhibitors analyzed. Although the drugs were assessed at uniform timepoints (baseline, week 4, and week 12), each biologic agent has a unique induction schedule and pharmacokinetic profile, which could have influenced early clinical outcomes. For instance, patients in the secukinumab group had received more cumulative doses by week 4 compared to those treated with bimekizumab. These differences should be considered when interpreting comparative efficacy results, especially in short-term assessments.

### 4.8. Future Perspectives

Exponential interpolation offers a visually intuitive and mathematically tractable method for approximating long-term treatment trends; nevertheless, it inherently assumes a consistent, monotonic rate of clinical improvement, which may not fully reflect the heterogeneous and often nonlinear nature of real-world disease progression. Exponential functions may oversimplify temporal dynamics by underestimating late plateau effects, treatment pauses, or flare-ups that are common in chronic immune-mediated diseases like psoriasis. Furthermore, our model does not account for adherence fluctuations or external modifiers (e.g., comorbidities, lifestyle changes), which may influence individual response patterns. As such, while exponential modeling serves as a useful exploratory tool, its generalizability to other cohorts should be approached with caution. Future research incorporating more flexible and individualized modeling techniques—such as mixed-effects models or machine learning-based trajectory clustering—could provide more nuanced insights and improve predictive validity across diverse populations. The integration of artificial intelligence to personalize exponential interpolation models at scale represents a promising future direction for treatment programs and real-world therapeutic monitoring, enabling dynamic, patient-specific predictions of clinical response across heterogeneous populations.

## 5. Conclusions

In this real-world cohort study, all evaluated IL-17 inhibitors—bimekizumab, ixekizumab, and secukinumab—demonstrated significant improvements in PASI, BSA, and DLQI scores. Bimekizumab showed numerically greater reductions in disease severity indicators, particularly during the early treatment phase, with the highest effect size observed at week 4. While exponential modeling of PASI, BSA, and DLQI trajectories suggested a higher probability of achieving complete clearance with bimekizumab, these differences did not reach statistical significance at the common endpoint of week 12.

Ixekizumab and secukinumab exhibited closely comparable clinical efficacy throughout the observation period, though subtle variations in response dynamics may relate to pharmacokinetics and baseline patient characteristics. Among patients with prior IL-23 inhibitor exposure and higher baseline disease burden, the initial response to bimekizumab appeared more pronounced; however, these subgroup findings require confirmation in larger, prospective studies.

Across all treatment groups, IL-17 inhibitors were well tolerated, with no serious adverse events or treatment discontinuations due to safety concerns. While these findings provide practical insights for biologic selection in moderate-to-severe plaque psoriasis, caution is warranted in interpretation due to sample size limitations, baseline variability, and differences in induction schedules. Future research incorporating larger, multicentric populations and individualized modeling techniques is warranted to further refine personalized therapeutic strategies.

## Figures and Tables

**Table 1:** Demographic and clinical characteristics of patients treated with bimekizumab, ixekizumab, and secukinumab. Values are presented as mean ± standard deviation or counts with corresponding percentages.

	Bimekizumab (*n*= 48)	Ixekizumab (*n* = 20)	Secukinumab (*n* = 30)
Sex	F: 17 (35.4%) M: 31 (64.6%)	F: 5 (25%) M: 15 (75%)	F: 9 (30%) M: 21 (70%)
Age [years]	43.8 ± 13.68	48.85 ± 11.82	47.2 ± 12.71
BMI [kg/m^2^]	29.38 ± 5.64	29.84 ± 5.37	30.36 ± 4.14
Years since psoriasis diagnosis [years]	19.88 ± 10.63	21.85 ± 10.61	20.5 ± 12.68
Co-occurrence of psoriatic arthritis	9 (18.75%)	6 (30%)	10 (30%)

**Table 2:** Comparative analysis of the efficacy of IL-17 inhibitors—bimekizumab, ixekizumab, and secukinumab—in reducing Psoriasis Area and Severity Index (PASI) scores after one month of treatment. Paired-sample t-tests confirmed statistically significant reductions for all three agents (*p* < 0.05). Effect sizes, calculated using Hedges’ g, illustrate the magnitude of PASI reduction in standard deviation units, providing a standardized measure of treatment efficacy.

	Timeline Between Doses—One Month	T-Statistic	Dof	*p*-Value	Eff. Size: Hedges-g
Bimekizumab	Between d1 and d2	16.672	47.000	<0.001	3.622
Ixekizumab	Between d1 and d3	6.653	19.000	<0.001	1.986
Secukinumab	Between d1 and d5	14.844	29.000	<0.001	2.813

**Table 3:** Incidence and types of adverse events observed among patients treated with IL-17 inhibitors. Data are presented as the number of patients and the percentage of the treatment group. No serious adverse events (SAEs) or therapy discontinuations due to AEs were reported.

Adverse Event	Bimekizumab (*n* = 48)	Ixekizumab (*n* = 20)	Secukinumab (*n* = 30)
Upper respiratory tract infections	2 (4.2%)	0 (0.0%)	3 (10.0%)
Oral candidiasis	1 (2.1%)	1 (5.0%)	0 (0.0%)
Urticaria	0 (0.0%)	2 (10.0%)	0 (0.0%)
Injection site reactions	0 (0.0%)	1 (5.0%)	0 (0.0%)
Total patients with any adverse event	3 (6.3%)	4 (20.0%)	3 (10.0%)
Serious adverse events	0 (0.0%)	0 (0.0%)	0 (0.0%)
Discontinuations due to AEs	0	0	0

## Data Availability

Reference to a dataset supporting results section: Kruczek, Wiktor (2025), “Interleukin-17 inhibitors in the treatment of moderate-to-severe psoriasis—real world data”, Mendeley Data, V1, doi: 10.17632/t8mnz5cyzy.1.

## Supplementary Materials

The following supporting information can be downloaded at: https://www.mdpi.com/article/10.3390/jcm14155421/s1, Table S1: Summary of interaction analyses between baseline variables (age, sex, PsA status) and treatment response.

## Abbreviations

The following abbreviations are used in this manuscript:

PASI	Psoriasis Area and Severity Index
BSA	Body surface Area
DLQI	Dermatology Life Quality Index
ADA	Anti-drug antibodies
IL-17/23	Interleukin-17/23
