# Supplementary material for: Comparative Analysis of the Long-Term Real-World Efficacy of Interleukin-17 Inhibitors in a Cohort of Patients with Moderate-to-Severe Psoriasis Treated in Poland
Source: J Clin Med. 2025 Aug 1;14(15):5421. doi: 10.3390/jcm14155421 (PMC12347414; doi:10.3390/jcm14155421)
Supplement: Supplementary file 1 [file jcm-14-05421-s001.zip › jcm-3765773-supplementary.pdf]

Supplementary Table S1 – confounding variables analysis

Biologic Agent	Dependent Variable	Covariate Tested	Significant Covariate Effects	Interaction Effect (Covariate × Dose)	Effect Size (η^2 dose)
Bimekizumab	PASI	BMI / Age / Sex / PsA	None	NS (p > 0.6)	0.872
Bimekizumab	BSA	BMI / Age / Sex / PsA	Age (significant BSA Δ2-3; trend p = 0.067)	Trend (p = 0.067)	0.786
Bimekizumab	DLQI	BMI / Age / Sex / PsA	None	NS (p > 0.2)	0.848
Ixekizumab	PASI	BMI / Age / Sex / PsA	None	NS (p > 0.2)	0.727
Ixekizumab	BSA	BMI / Age / Sex / PsA	None	NS (p > 0.1)	0.670
Ixekizumab	DLQI	BMI / Age / Sex / PsA	Sex (significant)	Significant (p = 0.022)	0.776
Secukinumab	PASI	BMI / Age / Sex / PsA	None	NS (p > 0.3)	0.880
Secukinumab	BSA	BMI / Age / Sex / PsA	None	NS (p > 0.5)	0.745
Secukinumab	DLQI	BMI / Age / Sex / PsA	PsA (significant)	Significant (p = 0.028)	0.759
